# Research and clinical implications of emerging evidence regarding patterns of postoperative opioid-induced respiratory depression

**DOI:** 10.17305/bb.2024.11123

**Published:** 2024-09-12

**Authors:** Toby N Weingarten, Atousa Deljou, Juraj Sprung

**Affiliations:** 1 Department of Anesthesiology and Perioperative Medicine, Mayo Clinic, Rochester, USA

**Keywords:** Apnea, postoperative, monitoring, study design

## Abstract

The wider availability of continuous respiratory monitors and advanced data abstraction techniques has led to a substantial increase in understanding of postoperative opioid-induced respiratory depression (OIRD), particularly regarding its incidence, presentation, temporal distribution, and risk factors. Self-limited episodes of OIRD are relatively common, typically presenting as repetitive apneas beginning in the postoperative period and continuing through the first night after surgery. In contrast, life-threatening episodes of OIRD are rare and usually occur on the day of surgery. Traditional monitoring of patient vital signs may be insensitive in detecting OIRD, while healthcare staff may be more adept at recognizing the concurrent development of somnolence. Although obstructive sleep apnea (OSA) is a known risk factor for OIRD, a more comprehensive phenotype is emerging-elderly patients with debility and substantial comorbidity. These advances have significant implications for managing postoperative OIRD. This review will focus on how these new insights into OIRD have highlighted knowledge gaps and created opportunities for future research and practice initiatives.

## Introduction

Critical life-threatening events of opioid-induced respiratory depression (OIRD) are severe complications that can lead to permanent morbidity and death [1]. These events have posed a persistent challenge for healthcare systems, prompting the development of practice initiatives [2–4], monitoring technologies [5–7], novel analgesic regimens [8, 9], and respiratory stimulants [10]. However, determining the effectiveness of any of these interventions in preventing critical OIRD events remains elusive, as traditional concepts regarding OIRD may be outdated or incorrect. In this review, we will examine the latest evidence on the incidence, phenotypic and temporal presentation, and risk factors for OIRD, as well as the implications of these new insights in guiding future research and shaping current practices. These concepts are summarized in Table 1.

**Table 1:** Implications of recent advances in postoperative OIRD on clinical research and practice

**The incidence of postoperative OIRD**• Self-limited episodes of OIRD occur in 1/3 – 1/2 of postoperative patients on general care wards• Incidence of severe OIRD* is approximately 1/1,000 postoperative admissions on general care wards
*Research implications*• Prospective trials • Powered to establish efficacy • Often biased to mitigate the risk of adverse events • Inadequately powered to detect severe OIRD events • Require quantification of self-limited OIRD episodes• Retrospective observational studies • Large cohorts are required to assess risks potentially associated with severe OIRD• Optimal alarm thresholds for continuous monitors have not been established
*Clinical implications*• Universal continuous monitoring for postoperative patients • Alarm fatigue occurs with a high rate of self-limited OIRD episodes • Optimal alarm thresholds for monitors may reduce alarm fatigue
**Presentation of OIRD**• Intermittent apneic episodes are the most dominant presentation of OIRD• Isolated bradypnea is very rare• Supplemental oxygen mitigates desaturation events secondary to hypoventilation• Healthcare providers often awaken sleeping patients before vital sign assessments • Can temporarily extinguish OIRD breathing patterns• Healthcare providers often do not recognize OIRD but do recognize somnolence
*Research implications*• The best modalities (capnography, pulse oximetry) to quantify OIRD episodes for prospective trials have not been established• The parameters of OIRD episodes have not been established
*Clinical implications*• Healthcare providers should be trained to assess respiratory efforts while patients are sleeping• Healthcare providers should be trained to recognize that somnolence can precede severe OIRD events
**Temporal distribution of OIRD**• Self-limited OIRD episodes begin in the PACU and continue onto the ward• The peak occurrence of self-limited OIRD is in the early morning hours• Severe OIRD episodes most commonly occur during the afternoon and evening following surgery
*Research implications*• Monitoring of postoperative OIRD should ideally extend beyond PACU admission• Interventions to prevent OIRD should continue past PACU admission
*Clinical implications*• Patients exhibiting OIRD during PACU stay should have an extended PACU admission• Ward nurses should be vigilant for severe OIRD during the first several hours after PACU discharge
**Risks for OIRD**• Obstructive sleep apnea is the most widely recognized risk factor for OIRD• Advanced age, debility and neurological disorders maybe more important risk factors• Increased risk with more extensive procedures and higher opioid dosages• Gabapentinoids and more sedating anesthetics increase OIRD risk
*Research implications*• Need to develop risk prediction scores for severe OIRD• Continuously assess emerging perioperative techniques for changes in OIRD risks
*Clinical implications*• Follow guidelines regarding the perioperative management of obstructive sleep apnea• Holistic assessment of surgical patients and adjust anesthetic management to mitigate risk• Appreciate PACU OIRD is strongly associated with severe OIRD on general care wards

*Severe OIRD was defined as the administration of naloxone to reverse opioid toxicity. OIRD: Opioid induced respiratory depression; PACU: Postanesthesia care unit.

### Incidence of postoperative OIRD: A common event albeit severe events are rare

#### Continuous monitoring and OIRD incidence

Over the past 15 years, various technologies capable of continuously monitoring and recording respiratory function data in the postoperative period have been studied. These studies consistently report high rates of postoperative respiratory depression events. The first large cohort study, conducted by Sun et al. [11], utilized pulse oximetry, blinded to healthcare providers, on 833 postoperative patients in general care wards. They found that 37% of subjects had clinically undetected hypoxemia (oxyhemoglobin saturation [SpO_2_] < 90% for ≥ 1 h). The PRediction of OIRD In patients monitored by capnoGraphY (PRODIGY) trial was a large multinational study of 1335 subjects that used both capnography and pulse oximetry, blinded to healthcare providers, on patients receiving opioid analgesics in general care wards. The study found that 46% of patients had at least one OIRD episode [5]. Measurement of respiratory effort, using noninvasive respiratory volume monitors that track changes in chest wall bioimpedance, indicated that approximately one-third of patients in general care wards experience OIRD [6]. Lastly, the use of acoustic respiratory rate monitors revealed that bradypnea occurred in 48% of postoperative patients in general care wards [7].

However, the clinical relevance of these OIRD episodes remains uncertain. Khanna et al. [12] found that PRODIGY patients who experienced OIRD episodes had longer hospital stays and higher hospital costs compared to those who did not. Yet, among the 614 patients in PRODIGY who experienced an OIRD episode, only seven had respiratory depression recognized by healthcare staff [13]. There is a possibility that a significant proportion of these postoperative OIRD episodes could be a component of underlying sleep breathing disorders, such as undiagnosed obstructive sleep apnea (OSA). OSA is a common but often undiagnosed comorbidity, with an estimated 20% of adult surgical patients not being preoperatively diagnosed with OSA [14]. The implications of this commonality can be illustrated by considering a postoperative patient with mild sleep apnea, diagnosed via polysomnography, who has an apnea-hypopnea index (AHI) of ten events per hour. Using the alarm settings from the PRODIGY trial [5], this patient would experience 80 respiratory events during 8 h of uninterrupted sleep. While these events may have long-term health consequences, such as an increased risk of developing hypertension, they are unlikely to significantly impact postoperative outcomes in the acute period.

#### Incidence of severe OIRD

Quantifying the incidence of life-threatening episodes of OIRD is more challenging because these events are rare. Acute respiratory failure requiring endotracheal intubation and the initiation of mechanical ventilation may result from opioid toxicity but can also arise from other etiologies, such as severe neurological disorders (e.g., stroke, encephalitis), hypoxemia secondary to ventilation-perfusion mismatch (e.g., pneumonia, pulmonary embolism), or other conditions (e.g., sepsis, multiorgan failure) [15]. A less sensitive but more specific marker of life-threatening OIRD is the administration of the opioid reversal drug naloxone [16]. A recent review reported that the incidence of postoperative naloxone administration in general care wards ranges from 3.7–53.3 per 10,000 patients [16]. An aggregate of these studies suggests an incidence of 1 emergency naloxone administration per 1000 postoperative patients admitted to general care wards. The rarity of severe respiratory events has important implications for OIRD research, which could lead to more precise clinical practice guidelines.

##### Implications for clinical research

Prospective randomized controlled trials are primarily conducted to assess the efficacy of an intervention, often with the goal of obtaining regulatory approval for a drug or device. However, a variety of methodological shortcomings typically limit the ability of such studies to quantify the risk of adverse events, particularly the low incidence of life-threatening OIRD events [17, 18]. Moreover, strict protocols designed to minimize the risk of adverse events, along with inconsistencies in reporting these events, represent additional limitations in these trials [17, 18]. Meta-analyses of such studies inherit these methodological limitations and may therefore underestimate the risk of OIRD [18].

###### Determining safety of analgesics

Perioperative gabapentinoids were advocated by practice guidelines as part of non-opioid multimodal analgesic protocols [19]. Since these drugs were considered to be “opioid-sparing,” they were theoretically expected to reduce the risk of serious OIRD events. Many studies on gabapentinoids were designed to minimize the risk of OIRD. For example, Hah et al. [20] conducted a prospective study on perioperative gabapentin use to assess postoperative pain resolution and opioid cessation. The exclusion criteria included patients with OSA, a known risk factor for postoperative OIRD [16]. Additionally, the study reported only 410 subjects, which is insufficient to reliably assess the risk of OIRD. Larger retrospective studies demonstrated a positive association between perioperative gabapentinoid use and serious episodes of OIRD [21–23], leading to a “black box warning” by the US Food and Drug Administration (FDA) regarding the risk of serious respiratory depression when gabapentinoids are used with other sedating medications [24].

More recently, there has been interest in perioperative methadone for analgesic therapy [25]. A recent report suggested that methadone use is not associated with an increased need for perioperative naloxone compared to conventional opioid analgesics [26]. In that study, 337 patients who were administered methadone were matched to 674 controls administered conventional opioids. The rate of postoperative naloxone administration was 2 (0.6%) in the methadone group and 1 (0.1%) in the conventional opioid group. The sample size in this study [26] is underpowered to assess the safety of methadone use in the perioperative period. It is important to note that the majority of FDA safety notices are issued after the regulatory process is completed [27]. Thus, it is incumbent upon perioperative physicians and healthcare systems to continually audit their practices to review serious OIRD events and assess whether there are changing trends in incidence or risk factors associated with novel drug practices.

###### Assessing efficacy of respiratory stimulants and novel opioids on OIRD incidence

There are several drugs that act as respiratory stimulants without reversing the analgesic effects of opioids, unlike the µ-antagonist naloxone [10]. For example, ENA001 (formerly known as GAL-021, a drug related to almitrine), is a selective antagonist of large-conductance big potassium channels in the carotid body chemoreceptors, which enhances hypoxic respiratory drive and has been shown to reverse alfentanil-induced OIRD without affecting its analgesic properties [28]. Please refer to van der Schrier et al. [10] for a comprehensive review of novel respiratory stimulants. Oliceridine is a unique opioid that induces µ-opioid receptor G-protein signaling, which provides analgesia while minimally activating the µ-receptor β-arrestin signaling pathway that is responsible for respiratory depression and gastrointestinal adverse effects [29]. Compared to morphine, oliceridine has been found to have a better therapeutic index, offering analgesia with fewer respiratory and gastrointestinal side effects [30, 31]. However, designing a study sufficiently powered to determine whether respiratory stimulants or novel opioids could reduce the incidence of severe OIRD in clinical settings would be prohibitive. For example, a study powered to detect a 50% reduction in naloxone administration would require approximately 50,000 subjects. Thus, alternative metrics to establish safety are required, but such metrics have not yet been established—a concept we will explore in the next section.

##### Implications for clinical practice

###### Postoperative monitoring strategies

To mitigate the risk of serious or fatal OIRD, there have been calls for universal monitoring of hospitalized post-surgical patients [32]. Why not use an oximeter on all patients so hospital staff can be alerted if a patient’s oxygen saturation is dropping? Pulse oximetry is widely available, inexpensive, and well understood by both laypeople and healthcare providers. A challenge to such an approach is defining clinically relevant thresholds for alarm activation. Sun et al. [11], using SpO_2_ ≤ 90% as a definition of hypoxemia, found that 37% of patients met this criterion. However, many of these patients were not in acute danger of respiratory arrest. Such overly sensitive settings lead to alarm fatigue, rendering the monitors less effective. This dilemma was addressed by McGrath et al. [33] with the introduction of the concept of “condition” and “surveillance” alarm thresholds. “Condition”-specific alarm thresholds are those used in high-acuity settings, such as during general anesthesia, where vital signs are closely monitored and small changes in patient physiology necessitate constant adjustments of anesthetic variables. In contrast, “surveillance”-specific alarm thresholds are more useful in lower-acuity settings. In one large academic hospital, all postoperative patients were continuously monitored with pulse oximetry using “surveillance”-specific alarm settings (SpO_2_ below 80% for 30 s), and when this threshold was reached, the automated system alerted the nurse via pager [4]. Using this algorithm, nurses received an average of four pages per patient per day. Questions about the clinical safety of these surveillance alarm settings were addressed by Taenzer et al. [4]. The primary outcomes of Taenzer’s study were rescue events and transfers to the intensive care unit before and after the implementation of monitoring changes. They found that the number of rescue events decreased from 3.4 to 1.2 per 1000 patient discharges, and intensive care unit transfers from 5.6 to 2.9 per 1000 patient days. The observed death rate after implementation was two, compared to four in the previous timeframe. Furthermore, McGrath et al. [33] retrospectively analyzed 6130 patients who received three days of continuously recorded SpO_2_ data while hospitalized in general care wards. They examined the effect of decreasing the alarm threshold from 88% to 80% and changing from an instant alarm to a 15-s delay. Using “surveillance” settings, there would be 2.5 pulse oximeter alarms per patient per day, compared to 148 alarms per patient per day with traditional “condition” settings [33].

Cutoffs for safe thresholds for continuous pulse oximetry monitoring to detect respiratory failure in general care wards have not been well defined in the literature. The unanswered question is whether “surveillance” settings should use universal thresholds or if a tailored approach based on specific patient comorbidities would be more appropriate. The latter approach would introduce an additional layer of clinical complexity. Furthermore, ideal thresholds for more advanced monitoring technologies, such as capnography, have not been established. For example, the threshold for apnea in the PRODIGY trial was based on definitions used for polysomnography [5]. However, as mentioned previously, a patient with underlying sleep apnea might frequently trigger the capnography alarm. The pattern of apneas leading to clinically relevant OIRD (e.g., the need for re/intubation, etc.) is not well understood. Is it the frequency of apneas, the duration of apneas, accompanying changes in pulse oximetry and heart rate, or a combination of these factors? Does it differ between patients with or without OSA? Given these unknowns, it remains unclear how this technology should be best utilized in hospital care wards.

### Presentation of OIRD: More than bradypnea

#### Presentation with continuous monitoring

**Figure 1.**
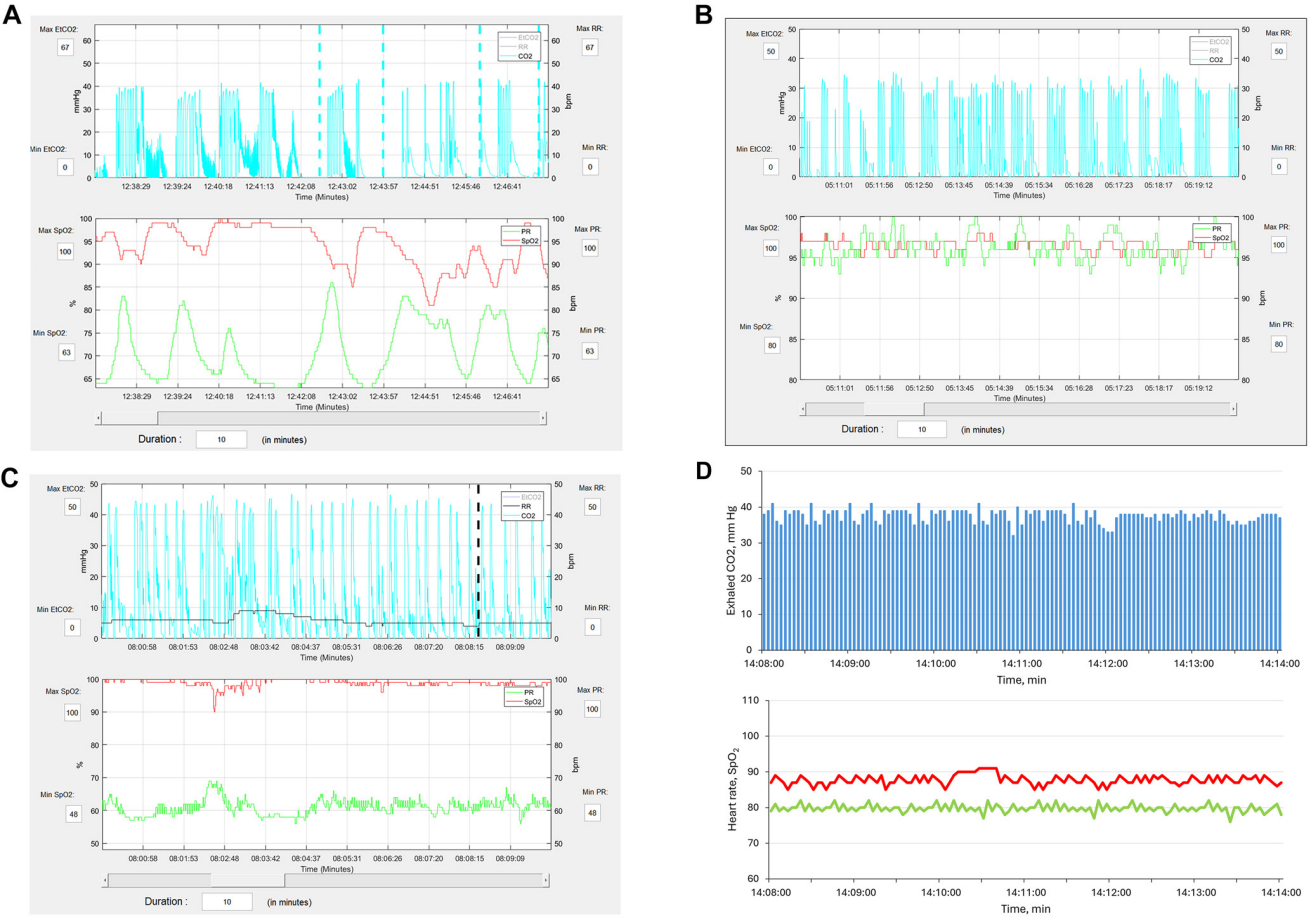
**Capnography and pulse oximetry patterns encountered during OIRD.** The top panels show measured exhaled CO_2_ (blue lines) over time in mmHg. The bottom panels show pulse oximetry oxyhemoglobin saturation (red line) and heart rate (green line). (A) This panel depicts the typical pattern of postoperative OIRD. The top panel shows pauses in respiratory effort, with the dashed blue lines indicating apneic episodes lasting ≥ 30 s. In this panel, the apneic episodes are accompanied by episodic decreases in oxyhemoglobin concentrations and increases in heart rate [40]. Reprinted with permission from Anesthesia & Analgesia and Wolters Kluwer Health, Inc. (B) The panel depicts typical postoperative OIRD in a patient receiving supplemental oxygen. The top panel exhibits intermittent apneic episodes, but in this case, changes in oxyhemoglobin concentration and heart rate are mitigated. Courtesy of the author’s work, data from the PRODIGY trial [5]. (C) The panel depicts a classic bradypnea pattern of postoperative OIRD. The top panel exhibits a slow and widened but stable exhaled CO_2_ pattern, and the black dashed line indicates a respiratory rate of fewer than 5 breaths per minute for ≥ 30 s. This patient is on supplemental oxygen; thus, oxyhemoglobin concentration remains in the normal range. This breathing pattern was present in less than 3% of respiratory depression patterns detected in the PRODIGY trial [5]. Courtesy of the author’s own work, data from the PRODIGY trial [5]. (D) The panel depicts a patient with normal respiratory effort but decreased oxyhemoglobin concentration. This pattern can emerge in patients with conditions such as atelectasis. Courtesy of the author’s work. OIRD: Opioid-induced respiratory depression.

Classic teaching about the effect of opioids on ventilation suggests that these drugs slow the respiratory rate and decrease sensitivity to arterial carbon dioxide and oxygen concentrations [34]. The PRODIGY trial [5] found that 97% of detected OIRDs presented as apnea and 58% as bradypnea. The typical pattern, shown in Figure 1A, involves intermittent apnea episodes interspersed with periods of normal respiratory effort. For patients on room air, the apneas are accompanied by episodic decreases in SpO_2_ and increases in heart rate. In contrast, for patients receiving supplemental oxygen, the intermittent apneas are often not associated with changes in SpO_2_ and heart rate (Figure 1B). Isolated bradypnea was observed in less than 3% of cases (Figure 1C). These observations are more consistent with opioids inducing pauses in ventilation through a combination of central apneas and obstructive apneas due to reduced hypoglossal nerve tone to the upper airway, causing obstructions during sleep. Another pattern of respiratory instability is isolated hypoxemia without signs of the decreased respiratory drive (Figure 1D), which would be more compatible with a pathological lung condition (e.g., pneumonia, atelectasis) resulting in a ventilation–perfusion mismatch.

#### Healthcare providers’ perception of OIRD

Analysis of how healthcare providers evaluate patients experiencing OIRD has yielded unexpected results. For example, Lee et al. [1] studied the American Society of Anesthesiology Closed Claim Database, which included 92 malpractice claims related to adverse outcomes from postoperative respiratory depression. Their study revealed that somnolence was recorded by nurses in 62% of cases prior to the adverse respiratory event. Additionally, “heavy snoring” was noted in 15% of cases, and somnolence accompanied snoring in 93% of those patients. Studies examining postoperative naloxone administration on general care wards also found that nurses frequently recorded somnolence or decreased levels of consciousness preceding severe respiratory events.

Weingarten et al. [35] examined postoperative naloxone administration in 134 patients on general care wards within the first 48 h of surgery and found that the indication for naloxone administration was excessive sedation in 43% of cases and respiratory depression in 52% of cases. Similarly, Deljou et al. [21] examined postoperative naloxone administration in 128 patients within the first 48 h of surgery and found that the indication for naloxone administration was excessive sedation in 44% of cases and respiratory depression in 56% of cases. Morales et al. [36] examined 162 medical and postsurgical patients who were administered naloxone and found that nurses noted neurologic signs (e.g., somnolence, decreased level of consciousness) in 80% of cases and pulmonary symptoms in 50% of cases.

There is a possibility that some cases of naloxone administration “to treat somnolence” were unrelated to OIRD, but it is also possible that patients with somnolence had unrecognized concurrent respiratory depression. This was illustrated in the PRODIGY trial, where a patient was administered naloxone for a “decreased level of consciousness” rather than respiratory depression, yet capnography identified eight apneic episodes immediately prior to the naloxone administration [13].

Examining respiratory drive requires a higher level of expertise and prolonged patient observation to differentiate between apneic spells and isolated bradypnea. In contrast, assessing the level of consciousness can be done quickly. This is especially true if respiratory assessment relies solely on pulse oximetry when the patient is on supplemental oxygen, which can mask decreases in oxyhemoglobin levels due to hypoventilation [37]. Additionally, if nurses awaken patients during the assessment, transitioning from an asleep to an awake state may terminate apnea spells. A recent simulation study [38] assessed how nurses performed postoperative vital checks and found that in 96% of assessments, the nurses woke patients prior to assessments, resulting in the missed detection of 85% of apnea spells occurring while the patient was asleep. 

##### Implications for clinical research

Life-threatening cases of postoperative OIRD are rare [16], making it challenging to construct adequately powered prospective studies on the safety of new analgesics or sedatives, the effectiveness of respiratory stimulants, or practice initiatives designed to mitigate the effects of OIRD associated with oversedation. The introduction of continuous monitoring technologies has provided numerous opportunities to assess respiratory status, including oxyhemoglobin saturation levels, respiratory rate, apnea spells, and minute ventilation. Each of these measurements has its advantages and limitations.

###### Pulse oximetry

Pulse oximetry provides data on oxyhemoglobin saturation and pulse rate. This technology can be utilized in various ways to assess interventions aimed at mitigating OIRD. Simplistically, one could calculate the duration that patients spend below a defined oxyhemoglobin saturation threshold. Another approach is to use pulse oximetry to calculate the oxygen desaturation index, which measures the number of desaturation events (defined as a 3% decrease in SpO_2_ for ≥ 10 s within a 120-s period) per hour. However, there are several limitations to either approach. Importantly, supplemental oxygen, commonly administered to postoperative patients, mitigates oxyhemoglobin desaturation in response to hypoventilation (Figure 1B) [37]. Thus, patients on supplemental oxygen may experience profound episodes of respiratory drive depression due to opioids and other sedatives, yet appear “normal” when assessed by pulse oximetry alone. Another major limitation is that common postoperative lung conditions, such as atelectasis, can lower oxyhemoglobin levels while preserving or even enhancing respiratory drive (hypoxemic drive). In this circumstance, an intervention designed to prevent respiratory depression may seem ineffective because oxyhemoglobin levels would be low despite adequate respiratory drive.

###### Advanced monitors

Other technologies, such as capnography, thoracic bioimpedance, and acoustic respiratory monitoring systems, measure respiratory drive by providing data on tidal volume, exhaled CO_2_, and respiratory rate. These technologies are often used alongside pulse oximetry to provide a holistic view of pulmonary function. However, they face ergonomic challenges. Capnography devices, typically incorporated into nasal cannulas, may interfere with eating, drinking, and speaking due to the sampling device extending from the nasal cannula to the mouth. While this may be manageable in a sedation setting (e.g., an endoscopy suite), it is less likely to be tolerated by awake patients on general care wards. Bioimpedance monitors can be dislodged by patient movement, and their adhesive backing can be undone by patient perspiration or chest hair. Lastly, acoustic respiratory monitors may be less accurate with facial hair and are prone to artifacts from patient movement and activities such as eating and speaking. Thus, the output from these monitors is complex and often requires time-intensive expert interpretation (author’s observations).

Furthermore, clinically relevant thresholds for respiratory depression have not been established, though definitions from sleep studies are often used (e.g., an apneic episode is defined as a period of apnea lasting ≥ 30 s) [5]. These monitors can capture respiratory depressive episodes when supplemental oxygen is used, even when pulse oximetry does not identify oxyhemoglobin desaturation, or when pulse oximetry indicates desaturation, but respiratory drive is preserved. However, how to quantify the effect of an intervention using such technologies has not yet been determined. Potential approaches include measuring the number of respiratory depressive events over time (akin to the AHI) or the total duration of respiratory drive instability. Nevertheless, such definitions require further research and the development of consensus.

##### Implications for clinical practice

###### Changing healthcare workers’ assessment of OIRD

The typical bedside assessment of respiratory status in hospitalized patients involves counting breaths to establish the respiratory rate, measuring oxyhemoglobin saturation with pulse oximetry, auscultating the chest, and asking the patient about symptoms of dyspnea [38]. We have observed that nurses usually awaken patients prior to these assessments [38]. This approach has notable flaws. Awakening a patient extinguishes the repetitive apneic breathing pattern typically associated with OIRD. Similarly, if patients with OIRD are awakened, their breathing may normalize temporarily, which can misleadingly be recorded as normal.

This practice can lead to problems when using continuous respiratory monitors. Specifically, in a sleeping patient with OIRD (or underlying sleep breathing disorders), continuous monitoring may trigger frequent alarms. However, each time the nurse assesses the patient by waking them, their respiratory status may seem reassuring. This can erode trust in the monitor, resulting in alarm fatigue. The adoption of “surveillance” monitoring strategies with less sensitive thresholds might reduce the frequency of alarms and only alert nurses to more serious episodes of respiratory depression [4]. However, it is not yet known whether this approach represents a safe clinical practice.

**Figure 2.**
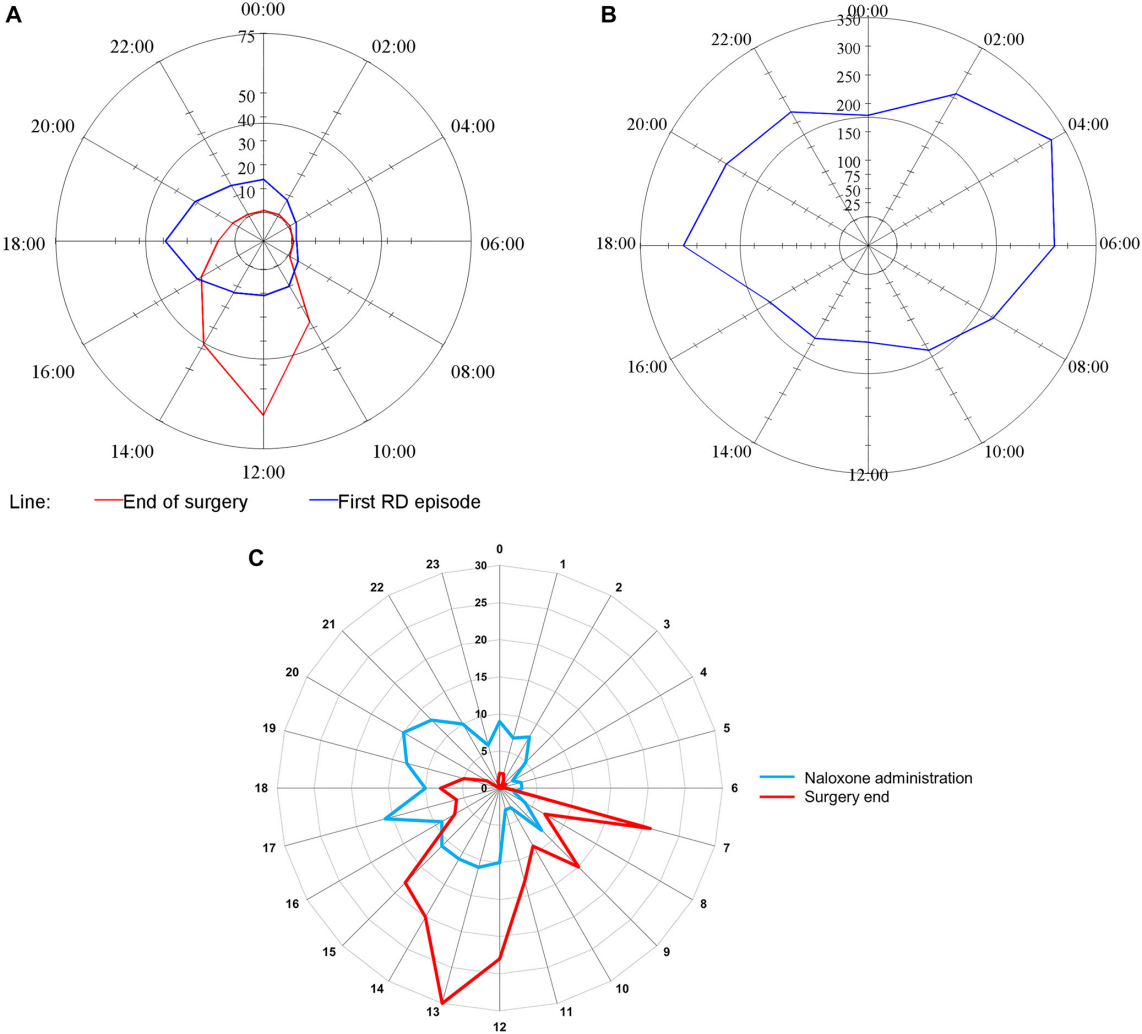
**Timing of opioid-induced respiratory depression episodes depicted on 24-h clocks.** The magnitude of each spoke is the total number of events between the previous spoke time and the current spoke time (e.g., at 02:00, the number of episodes that occurred between 00:00 and 02:00). The red line indicates the end of the surgery, and the blue line indicates respiratory depressive episodes. The scale of episodes is different between the three plots [40]. Reprinted with permission from Anesthesia & Analgesia and Wolters Kluwer Health, Inc. (A) The time of day for the first detected episode of respiratory depression among a subset of patients from the PRODIGY trial [40]. The majority of initial episodes occurred in the afternoon and evening following surgery. Reprinted with permission from Anesthesia & Analgesia and Wolters Kluwer Health, Inc. (B) The concentration of all respiratory depressive episodes among a subset of patients from the PRODIGY trial [40]. There is a bilobed distribution with many episodes occurring in the afternoon and evening, but the majority of episodes occurring in the early morning hours. Reprinted with permission from Anesthesia & Analgesia and Wolters Kluwer Health, Inc. (C) The time of day naloxone was administered on general care wards following surgery from two studies of postoperative naloxone administration from Mayo Clinic [16]. The majority of naloxone administrations occurred in the afternoon and evening following surgery. Reprinted with permission from International Anesthesiology Clinics and Wolters Kluwer Health, Inc.

To reiterate the above-discussed important concept, the American Society for Pain Management Nursing Guidelines clearly recommends that nurses quietly observe a sleeping patient’s breathing pattern for at least 1 min, looking for signs of airway obstruction or apneas [39]. This practice would increase the likelihood of nursing staff detecting OIRD. However, this approach is not widely practiced [38] and needs to be emphasized in nursing training and clinical practice by nurse managers. Even so, busy healthcare providers may not have adequate time to observe a sleeping patient for several minutes to conclude whether apneic episodes are occurring. Current practices seem adept at identifying decreased levels of consciousness, which often precede critical respiratory events [1], so nursing guidelines should emphasize that such patients should undergo more frequent assessments of respiratory status.

### Temporal distribution of OIRD: Dead at dinnertime

Data from the capnography and pulse oximetry patterns in the PRODIGY trial [5] provided a unique opportunity to determine the timing of postoperative OIRD. In the initial PRODIGY study, patients were categorized into two groups: those with OIRD and those without. This approach focused only on the first adjudicated OIRD episode due to the overwhelming number of detected episodes. However, in Driver et al. [40], all capnography and pulse oximetry data from two PRODIGY sites were closely analyzed. The authors found that among patients who experienced OIRD, 87.7% had multiple episodes. They were also able to construct detailed maps of when the initial event occurred (Figure 2A) and the concentration of all events (Figure 2B). The initial OIRD events were detected in the late afternoon and early evening (Figure 2A), though it is important to note that patients did not have capnography or pulse oximetry data during their postoperative care unit (PACU) stay and transport from the PACU to the ward. Additionally, there was a bilobed concentration of all events, with one peak in the late afternoon/early evening and a larger, significant peak during the early morning hours (Figure 2B). These early morning events have been postulated to be more clinically important [41] because patients are less closely monitored, and thus respiratory instability is more likely to progress to a critical level. However, data from the Mayo Clinic indicate that the most frequent time of naloxone administration on the surgical wards is in the late afternoon/early evening on the day of surgery (Figure 2C) [16].

Interestingly, Mayo Clinic naloxone data also indicated that patients who have PACU episodes of respiratory depression are five times more likely to receive naloxone in the general care wards [21, 35]. Another study that used bioimpedance monitors on patients in the PACU, and then for 12 h on the wards, found that those patients who experienced respiratory depression in the last 30 min of the PACU admission continued to have episodes of respiratory depression for hours on the wards compared to their controls [42]. These observations suggest that residual sedation from anesthesia and opioids during surgery and PACU admission may place vulnerable patients at increased risk for critical events at the beginning of their general care ward admission.

#### Implications for clinical research

Clinical studies examining the effects of novel respiratory stimulants [10] and opioids with fewer effects on respiratory drive [29–31] need to consider these temporal observations when determining the duration of postoperative monitoring. Ideally, the administration of respiratory stimulants would be initiated in the PACU and continue through the postoperative period until the following morning. Likewise, continuous monitoring for episodes of OIRD should extend beyond the PACU admission and continue onto the general care wards through the night, until the patient awakens the next day. Similarly, studies examining mitigation practices should follow a similar timeline.

#### Implications for clinical practice

Understanding the temporal patterns of postoperative OIRD has several practical implications that can be easily integrated into the daily routines of healthcare staff. First, the PACU stay of any patient experiencing an OIRD episode should be extended. For example, the Mayo Clinic extends the PACU stay by at least 1 h for any patient who exhibits OIRD during anesthesia recovery [3]. Second, the PACU staff should alert the accepting ward if a patient has an episode of OIRD so that heightened vigilance for this complication can be maintained. Additionally, the ward staff should be educated that patients are prone to decompensation early in their ward admission, and continuous monitors should be applied upon admission rather than waiting until the patients are about to sleep.

### Risks for OIRD: Beyond OSA and opioids

OSA is the most recognized risk factor for postoperative respiratory complications and is the focus of several prominent perioperative management guidelines [2, 19, 43]. However, when considering the risk for OIRD, it is important to look beyond OSA and adopt a more holistic approach to assessing patients. The PRODIGY trial found that advancing age is associated with OIRD risk, with an odds ratio of 2.2 for ages 60–70 years; 3.4 for ages 70–80 years; and 4.8 for patients over 80 years old [5]. Furthermore, studies that used naloxone administration as a surrogate for severe OIRD found a four-fold increase in risk among patients with underlying neurodegenerative conditions [35] and frail patients [21]. These two studies also found a two-fold increased risk for OIRD among patients with OSA [21, 35]. Moreover, cardiopulmonary diseases, other than sleep breathing disorders, have also been found to be associated with increased OIRD risk [5, 35].

Not surprisingly, more extensive surgeries and more sedating anesthetic regimens are associated with OIRD. As expected, intraoperative opioids, especially longer acting and higher doses, are linked to an increased risk of OIRD [44–46]. The intrathecal administration of hydrophilic opioids (morphine, hydromorphone) is also known to increase the risk for postoperative OIRD, which may have a delayed onset, necessitating prolonged monitoring [47, 48]. The risk also increases with the use of more soluble halogenated volatile anesthetic gases [49, 50], the routine administration of benzodiazepines [49], and gabapentinoids [21–23, 44, 46, 51, 52]. Lastly, the PACU course can yield important information regarding OIRD risk on the wards, specifically patients who experience episodes of respiratory depression in the PACU are at increased risk for naloxone administration after PACU discharge (see next section) [21, 35].

#### Implications for clinical research

The PRODIGY study developed a PRODIGY score that can be used to categorize patients into low, intermediate, and high risk for postoperative OIRD as detected by capnography and/or pulse oximetry [5]. However, as mentioned earlier, almost half of the PRODIGY cohort developed postoperative OIRD. The PRODIGY score has never been validated for severe OIRD episodes (e.g., patients requiring naloxone therapy), but given the commonality of self-limited OIRD and the rarity of life-threatening OIRD, the PRODIGY score may be limited by being overly sensitive, with a lack of specificity to identify patients at risk for serious OIRD.

Electronic medical records now serve as vast repositories of clinical data and offer powerful data extraction capabilities. Statistical software can leverage this information to develop improved prediction tools for rare clinical events, such as serious postoperative OIRD. Additionally, these data repositories should be examined to assess the safety of anesthetic management and emerging trends, such as the perioperative administration of methadone for non-cardiac surgeries [25].

Results from retrospective studies have found strong associations between various aspects of anesthetic care and postoperative OIRD [21, 46], and changes in anesthetic management have been associated with a reduction in the incidence of postoperative OIRD [49]. These findings support the need for robust prospective trials to assess whether various anesthetic techniques can reduce the incidence of OIRD. For example, ultrashort-acting remifentanil and remimazolam are attractive agents to investigate as components of anesthetic management that may reduce OIRD risk.

#### Implications for clinical practice

Efforts by medical societies to prioritize OSA in mitigating the risk of OIRD are commendable [2, 19, 43]. However, clinicians should take a holistic view of surgical patients and recognize the contributions of advancing age, neurodegenerative conditions, debility, and other disease processes to OIRD risk. Patients undergoing therapy for OSA should continue this therapy in the postoperative period. Furthermore, anesthetic regimens for high-risk patients should be modified to promote more rapid anesthetic recovery by utilizing less soluble anesthetics and minimizing the use of sedating analgesic medications [49]. Regional anesthetic techniques should be employed for high-risk patients, if feasible [46].

**Figure 3.**
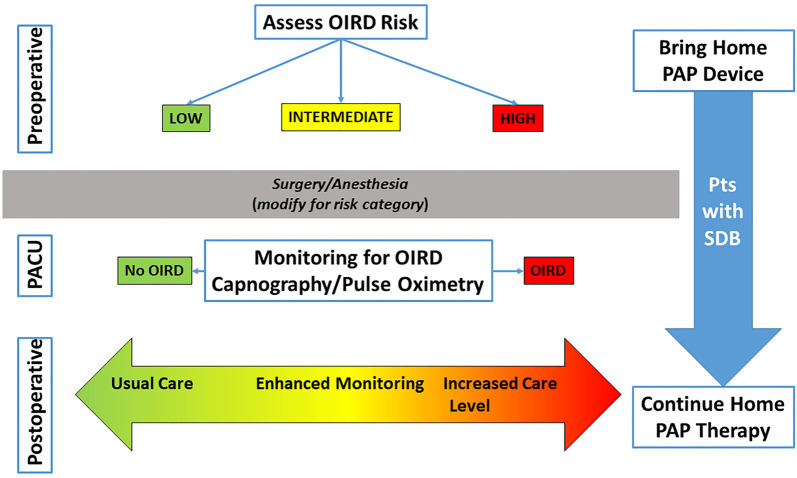
**Proposed clinical pathway for patients with postoperative OIRD.** Clinical decisions regarding the postoperative level of care are complex and unique for each patient. Preoperatively, patients should undergo a risk assessment for respiratory depression. The surgical and anesthetic management should be tailored to this risk. During anesthesia recovery, patients’ respiratory status should be monitored for various signs of respiratory depression. Postoperative management decisions regarding the level of monitoring and care should be guided by preoperative status, intraoperative events, and the anesthesia recovery course. Home therapies for sleep-disordered breathing should be continued into the postoperative period. PACU: Postanesthesia care unit; OIRD: Opioid-induced respiratory depression; SDB: Sleep-disordered breathing; PAP: Positive airway pressure [55]. Reprinted with permission from Anesthesiology and Wolters Kluwer Health, Inc.

Because the onset of OIRD can be delayed following the intrathecal administration of hydrophilic opioids, patients should be monitored with continuous pulse oximetry for the first 24 postoperative hours [53]. Recent evidence has cast doubt on the analgesic efficacy of perioperative gabapentinoids [54], and given their propensity to contribute to OIRD [21–23], these medications should no longer be used during the perioperative period. Clinicians should carefully assess patients experiencing respiratory depression during anesthesia recovery. The anesthesiologist should consider the entire perioperative course—from preoperative patient assessment to the PACU course—in determining the optimal postoperative care plan prior to PACU discharge (the proposed pathway is summarized in Figure 3) [55].

## Conclusion

Postoperative OIRD is a common complication, typically presenting as repetitive apneic episodes that first develop during anesthesia recovery and may continue through the first postoperative night. Although rare, self-limited episodes of OIRD can deteriorate into life-threatening respiratory failure, leading to serious morbidity and even mortality. When OIRD is used as a research endpoint, investigators need to recognize the continuum of OIRD severity to set appropriate definitions and ensure that studies are adequately powered. Similarly, clinical practices must account for these nuances of postoperative OIRD to develop the best perioperative care and postoperative monitoring strategies. Finally, clinical outcomes must be continuously reviewed to identify emerging trends and changes in OIRD incidence.
